# A first-in-human phase 1 study of nofazinlimab, an anti-PD-1 antibody, in advanced solid tumors and in combination with regorafenib in metastatic colorectal cancer

**DOI:** 10.1038/s41416-023-02431-7

**Published:** 2023-09-20

**Authors:** Daphne Day, John J. Park, Jermaine Coward, Ben Markman, Charlotte Lemech, James C. Kuo, Amy Prawira, Michael P. Brown, Sarwan Bishnoi, Dusan Kotasek, R. Matthew Strother, Rasha Cosman, Rila Su, Yiding Ma, Zenglian Yue, Hui-han Hu, Rachel Wu, Peiqi Li, Archie N. Tse

**Affiliations:** 1 Department of Medical Oncology, Monash Health, Clayton, VIC Australia; 2 Faculty of Medicine, Nursing and Health Sciences, Monash University, Clayton, VIC Australia; 3 Department of Clinical Medicine, Faculty of Medicine, Health and Human Sciences, Macquarie University, North Ryde, NSW Australia; 4 Medical Oncology, Icon Cancer Care - South Brisbane, South Brisbane, QLD Australia; 5 Faculty of Medicine, University of Queensland, Herston, QLD Australia; 6 Drug Development, Scientia Clinical Research, Randwick, NSW Australia; 7 School of Clinical Medicine, University of New South Wales, Sydney, NSW Australia; 8 Department of Medical Oncology, The Kinghorn Cancer Centre, St. Vincent’s Hospital Sydney, Darlinghurst, NSW Australia; 9 Cancer Clinical Trials Unit, Royal Adelaide Hospital, Adelaide, SA Australia; 10 School of Medicine, University of Adelaide, Adelaide, SA Australia; 11 Medical Oncology, Ashford Cancer Centre Research and ICON Cancer Centre, Kurralta Park and Division of Medicine, University of Adelaide, Adelaide, SA Australia; 12 Medical Oncology, Christchurch Hospital, Christchurch, Canterbury, New Zealand; 13 Translational Medicine and Early Development, CStone Pharmaceuticals (Suzhou) Co., Ltd., Suzhou, China; 14 Clinical Department, CStone Pharmaceuticals (Suzhou) Co., Ltd., Suzhou, China

**Keywords:** Cancer immunotherapy, Phase I trials

## Abstract

**Background:**

We assessed nofazinlimab, an anti-PD-1 antibody, in solid tumors and combined with regorafenib in metastatic colorectal cancer (mCRC).

**Methods:**

This phase 1 study comprised nofazinlimab dose escalation (phase 1a) and expansion (phase 1b), and regorafenib dose escalation (80 or 120 mg QD, days 1–21 of 28-day cycles) combined with 300-mg nofazinlimab Q4W (part 2a) to determine safety, efficacy, and RP2D.

**Results:**

In phase 1a (*N* = 21), no dose-limiting toxicity occurred from 1 to 10 mg/kg Q3W, with 200 mg Q3W determined as the monotherapy RP2D. In phase 1b (*N* = 87), 400-mg Q6W and 200-mg Q3W regimens were found comparable. In part 2a (*N* = 14), both regimens were deemed plausible RP2Ds. Fatigue was the most frequent treatment-emergent adverse event (AE) in this study. Any-grade and grade 3/4 nofazinlimab-related AEs were 71.4% and 14.3%, 56.3% and 5.7%, and 57.1% and 21.4% in phases 1a, 1b, and part 2a, respectively. ORRs were 14.3% and 25.3% in phases 1a and 1b, respectively. In part 2a, no patients had radiological responses.

**Conclusions:**

Nofazinlimab monotherapy was well tolerated and demonstrated preliminary anti-tumor activity in multiple tumor types. Regorafenib plus nofazinlimab had a manageable safety profile but was not associated with any response in mCRC.

**Clinical trial registr ation:**

Clinicaltrials.gov (NCT03475251).

## Background

The cell surface receptor programmed cell death protein-1 (PD-1) plays an important role in down-regulating the immune system and suppressing T cell activity [1–4]. Its ligand, programmed death ligand-1 (PD-L1), is highly expressed in several human cancers. The inhibition of PD-1 and PD-L1 pathways has emerged as one of the most effective therapeutic strategies in various cancers [5] and several antibodies targeting PD-1 or its ligand, PD-L1 have been approved to treat various solid and hematologic malignancies [6–11].

Nofazinlimab (formerly CS1003) is a humanized, recombinant immunoglobulin G4 anti-PD-1 monoclonal antibody (mAb). Nofazinlimab is one of a few anti-PD-1 antibodies that can recognize both human and murine PD-1. The binding potency of nofazinlimab to human PD-1 was comparable to that of pembrolizumab (K_D_: 6.13 nM vs 2.59 nM) [12]. Furthermore, nofazinlimab exhibited favorable toxicology, pharmacology, and safety profiles in preclinical experiments [12].

Regorafenib is an oral multi-kinase inhibitor that has demonstrated modest activity and survival gains in metastatic colorectal cancer (mCRC) after progression on standard-of-care therapies, including fluoropyrimidine-, oxaliplatin-, and irinotecan-based chemotherapy, anti-vascular endothelial growth factor therapy, and, in the case of KRAS wildtype tumors, anti-epidermal growth factor receptor therapy [13, 14]. In murine models, the combination of regorafenib plus anti-PD-1 mAb exhibited superior tumor growth suppression compared with either treatment alone [15, 16]. Encouraging preliminary results have been observed with the combination of nivolumab, an anti-PD-1 antibody, and regorafenib in patients with microsatellite stable (MSS) or mismatch repair–proficient advanced gastric cancer and mCRC in a phase 1b study [14, 17]. Given the lack of effective treatment options in refractory mCRC, nofazinlimab in combination with regorafenib was explored in the present study.

Here we report the findings of the first-in-human phase 1 study of nofazinlimab, in monotherapy in patients with solid tumors, and in combination with regorafenib in patients with mCRC (NCT03475251).

## Methods

### Study design

This was a phase 1, open-label, dose-escalation and dose-expansion study conducted from 4 May 2018 to 31 May 2021 at eight sites in Australia and one site in New Zealand. The study was approved by independent ethics committee/institutional review boards at each site, and adhered to the Declaration of Helsinki and Good Clinical Practice guidelines. All patients provided written informed consent.

Two parts were planned: part 1 (monotherapy dose escalation [phase 1a] and dose expansion in select tumor types [phase 1b]) and part 2 (nofazinlimab in combination with regorafenib dose escalation [part 2a] in mCRC and expansion [part 2b]) (Supplementary Fig. 1). Dose escalation followed a modified 3 + 3 scheme with 3 to 6 patients enrolled in sequential cohorts. In part 1a, nofanzilimab was administered intravenously once every 3 weeks (Q3W) at sequential dose levels of 1 mg/kg, 3 mg/kg, 200 mg (fixed dose) and 10 mg/kg. The DLT assessment period was the first treatment cycle in part 1 phase 1a, and part 2a. DLT definitions are detailed in the Supplementary Materials. The Safety Monitoring Committee determined a preliminary recommended phase 2 dose (RP2D) according to the safety, pharmacokinetics (PK), tolerability, and preliminary anti-tumor activities of nofazinlimab observed in the part 1 phase 1a stage and other available data. Once the preliminary RP2D was determined, additional patients were to be enrolled at this dose level up to a total of 10 patients.

In part 1 phase 1b, patients received nofazinlimab at 200 mg Q3W in arms 1 and 2, and were alternately enrolled to receive nofazinlimab at 200 mg Q3W in arm 3 or 400 mg Q6W in arm 4 (*n* = 20–30 per arm).

In part 2a, nofazinlimab was administered intravenously at 300 mg Q4W combined with regorafenib orally at 80 mg (dose level 1) and 120 mg (dose level 2) once daily (QD) for the first 21 days of each 28-day cycle. The regorafenib label dose is 160 mg taken orally QD for the first 21 days of each 28-day cycle. Based on the regorafenib registration study results of CONCUR and CORRECT, most of grade 3/4 AEs (54% patients in 160 mg regorafenib treatment developed grade 3/4 AE) occurred in the first two cycles [14, 18]. In the phase I study of nivolumab and regorafenib in Japanese patients with advanced or metastatic CRC, dose escalation safety data showed that 160 mg regorafenib was not tolerated (three DLTs in three patients), while 120 mg or 80 mg regorafenib combination with nivolumab had no DLTs [17]. Therefore, the doses of 80 mg and 120 mg QD for regorafenib were selected for the combination with nofazinlimab for part 2. If these dose regimens were considered intolerable, other dosing schedules planned would proceed (see Supplementary Materials). The detailed dose-escalation plan is provided in the Supplementary Materials. The schedule of 300 mg Q4W for nofazinlimab was studied in part 2 to allow synchronization with that of regorafenib and considered safe based on the safety and PK data from part 1 arm 4 (400 mg Q6W), which would cover the maximum observed concentration (C_max_) exposure of the 300 mg Q4W schedule.

The primary objectives were to evaluate the safety, tolerability, maximum tolerated dose (MTD), if any, and RP2D of nofazinlimab in part 1 phase 1a and regorafenib in combination with nofazinlimab in part 2a. The primary objective of part 1 phase 1b was to evaluate the objective response rate (ORR) of two dosing schedules of nofazinlimab in patients with selected tumor types. Secondary objectives included assessing the PK, preliminary anti-tumor activity and immunogenicity of nofazinlimab. Exploratory objectives included biomarker analyses such as PD-L1 expression.

Patients could receive treatment for up to 2 years or until treatment discontinuation due to disease progression, patient withdrawal or significant adverse events (AEs) (as defined in the protocol). Patients who were still benefiting from the study drug after the completion of the main study were switched to the extension study phase.

### Patients

Part 1 included patients with histologically or cytologically confirmed advanced or metastatic (unresectable), relapsed, or refractory solid tumors who had failed/did not tolerate standard therapy or for whom there was no available standard treatment. Selected tumor types in part 1 phase 1b included the following: arm 1—soft tissue sarcoma, including, but not limited to, undifferentiated pleomorphic sarcoma and dedifferentiated or other high-grade liposarcomas; arm 2—malignant pleural mesothelioma; arms 3 and 4—bladder cancer, Merkel cell carcinoma, gastric cancer, esophageal carcinoma, small-cell lung cancer, large-cell lung cancer, head and neck squamous cell carcinoma, or cutaneous squamous cell carcinoma; and any solid tumors with microsatellite instability-high or deficient mismatch repair (MSI-H/dMMR). Patients in part 2 had mCRC and had progressed or intolerable toxicities after least two lines of standard-of-care therapies (i.e., fluorouracil, oxaliplatin, and irinotecan-based chemotherapy). Patients with known MSI-H/dMMR and patients who had previously received regorafenib, fruquintinib or other VEGFR tyrosine kinase inhibitors were excluded from part 2. All patients had an Eastern Cooperative Oncology Group performance status of 0 or 1 and a life expectancy ≥3 months. Patients who previously received any targeted T-Cell co-regulated proteins (immune checkpoint proteins) antibody/medicine (including PD-1 and PD-L1) were excluded. Detailed inclusion and exclusion criteria are provided in the Supplementary Materials.

### Safety and tolerability

Toxicity or AEs were graded using National Cancer Institute-Common Terminology Criteria for AEs (NCI-CTCAE) version 4.03. The mandatory safety follow-up visits were conducted 30, 60, and 90 days (±3 days) after the last dose of study therapy. Patients who discontinued study treatment due to intolerable toxicity were followed until improvement or resolution to grade 0 or 1.

### Efficacy

Efficacy endpoints included ORR (complete response [CR] + partial response [PR]); disease control rate (DCR) (CR + PR + stable disease); progression-free survival (PFS); overall survival (OS); and duration of response (DOR). Disease assessment by radiographic imaging (computed tomography or magnetic resonance imaging) was performed and recorded by investigator per schedule (as detailed in the Supplementary Materials), according to RECIST version 1.1.

### Pharmacokinetics

PK parameters for nofazinlimab were evaluated at pre-infusion, end-of-infusion, and end-of-infusion +30 min/+90 min/+6 h/+24 h/+72 h/+ 168 h/+ 336 h in cycles 1 and 4 and pre-dose in subsequent cycles. For regorafenib, blood samples were collected at 0 h, 3 h, 6 h and 24 h on cycle 1 days 1 and 21. Samples were processed and analyzed at a central laboratory.

PK parameters evaluated included area under the concentration–time curve from the time of dosing to day 21 (AUC_0-21d_), area under the concentration–time curve to infinite time (AUC_0-∞_), C_max_, time to maximum observed serum concentration, half-life, clearance, and volume of distribution at steady state of nofazinlimab; and the C_max_ and minimum observed concentration after the administration of a given dose (C_min_) of regorafenib.

### Biomarkers

As an exploratory endpoint, PD-L1 expression in archival or fresh biopsy tumor tissues collected at baseline was assessed centrally by immunohistochemistry (Ventana PD-L1 SP263) for patients enrolled in part 1 phase 1b. The expression was scored as the percentage of tumor cells (TC%) or tumor-infiltrating immune cells (IC%) with positive staining of PD-L1. The relationships between PD-L1 expression, tumor response, and PFS were evaluated.

### Statistical methods

Details of sample sizes for each part and definitions of analysis sets are provided in the Supplementary Materials. Categorical data were summarized using frequencies and percentages (*n*, %) in each category, and continuous data were summarized with descriptive statistics. For the efficacy analysis, ORR and DCR were estimated along with 95% confidence intervals (CIs) using the Clopper–Pearson method. Kaplan–Meier method was used to estimate median PFS, PFS rates, median OS, OS rates, and median DOR, and their 95% CIs were evaluated using the Brookmeyer–Crowley methodology. Imputation for missing results was not performed.

The relationships between PD-L1 expression (TC% and IC%), tumor response and PFS were presented for evaluable patients by comparing patients with TC ≥ 1% versus TC < 1% and patients with IC ≥ 1% versus IC < 1%.

PK parameters were derived using Phoenix WinNonlin version 8.2 (Certara USA Inc, Princeton, NJ, USA). All statistical analyses were performed using SAS software version 9.4 or higher (SAS Institute Inc., Cary, NC, USA).

## Results

### Patients

In total, 122 patients received treatment on study and all patients were included in the safety and efficacy analysis sets. Median (range) follow-up was 28.6 (0.9–29.0) months for part 1 phase 1a, 17.9 (0.9–24.8) months for part 1 phase 1b, and 6.7 (1.8–13.6) months for part 2a. Median (range) treatment durations of nofazinlimab were 15.0 (3.0–126.3) weeks for part 1 phase 1a, 18.0 (3.0–108.0) weeks for part 1 phase 1b, and 8.2 (4.0–56.1) weeks for part 2a.

The baseline demographic and clinical characteristics of the study patients are summarized in Table 1. In part 1 phase 1a, 21 patients received nofazinlimab across four dose-escalating cohorts (1 mg/kg, *n* = 3; 3 mg/kg, *n* = 5; 200 mg fixed dose, *n* = 8; and 10 mg/kg, *n* = 5). In part 1 phase 1b, 87 patients with selected tumor types received nofazinlimab across four arms: (arm 1, *n* = 20; arm 2, *n* = 7; arm 3, *n* = 29; and arm 4, *n* = 31). Median (range) age was 63.0 (21–83) and 65.0 (26–85) years in phases 1a and 1b, respectively. More than half of the patients (61.9% in phase 1a and 62.1% in phase 1b) were male and the majority of the patients were white (90.5% in phase 1a and 92.0% in phase 1b).

**Table 1:** Baseline demographic and clinical characteristics.

	Part 1 phase 1a *N* = 21	Part 1 phase 1b nofazinlimab monotherapy					Part 2a nofazinlimab combination (nofazinlimab 300 mg Q4W)		
		Arm 1 200 mg Q3W *n* = 20	Arm 2 200 mg Q3W *n* = 7	Arm 3 200 mg Q3W *n* = 29	Arm 4 400 mg Q6W *n* = 31	Total *N* = 87	Regorafenib 80 mg *n* = 7	Regorafenib 120 mg *n* = 7	Total *N* = 14
Sex									
Male	13 (61.9)	10 (50.0)	4 (57.1)	18 (62.1)	22 (71.0)	54 (62.1)	4 (57.1)	3 (42.9)	7 (50.0)
Female	8 (38.1)	10 (50.0)	3 (42.9)	11 (37.9)	9 (29.0)	33 (37.9)	3 (42.9)	4 (57.1)	7 (50.0)
Age, years	63.0 (21, 83)	57.0 (26, 85)	70.0 (64, 76)	64.0 (36, 84)	68.0 (37, 83)	65.0 (26, 85)	56.0 (37, 70)	44.0 (37, 64)	49.0 (37, 70)
Race									
White	19 (90.5)	19 (95.0)	7 (100.0)	27 (93.1)	27 (87.1)	80 (92.0)	5 (71.4)	6 (85.7)	11 (78.6)
Asian	2 (9.5)	0	0	1 (3.4)	4 (12.9)	5 (5.7)	1 (14.3)	0	1 (7.1)
Other	0	1 (5.0)	0	1 (3.4)	0	2 (2.3)	1 (14.3)	1 (14.3)	2 (14.3)
ECOG PS									
0	11 (52.4)	14 (70.0)	3 (42.9)	14 (48.3)	18 (58.1)	49 (56.3)	4 (57.1)	5 (71.4)	9 (64.3)
1	10 (47.6)	6 (30.0)	4 (57.1)	15 (51.7)	13 (41.9)	38 (43.7)	3 (42.9)	2 (28.6)	5 (35.7)
MSI-H/dMMR									
Yes	2 (9.5)	1 (5.0)	0	5 (17.2)	5 (16.1)	11 (12.6)	0	0	0
No	11 (52.4)	2 (10.0)	1 (14.3)	7 (24.1)	9 (29.0)	19 (21.8)	6 (85.7)	4 (57.1)	10 (71.4)
Unknown	8 (38.1)	17 (85.0)	6 (85.7)	17 (58.6)	17 (54.8)	57 (65.5)	1 (14.3)	3 (42.9)	4 (28.6)
Patients with at least one KRAS/NRAS/BRAF mutation	—	—	—	—	—	—	5 (71.4)	2 (28.6)	7 (50.0)
KRAS mutation	—	—	—	—	—	—	3 (42.9)	2 (28.6)	5 (35.7)
NRAS mutation	—	—	—	—	—	—	2 (28.6)	0	2 (14.3)
BRAF mutation	—	—	—	—	—	—	0	1 (14.3)	1 (7.1)
Cancer stage at screening									
Stage III	0	0	6 (85.7)	2 (6.9)	3 (9.7)	11 (12.6)	0	0	0
Stage IV	20 (95.2)	20 (100.0)	1 (14.3)	25 (86.2)	25 (80.6)	71 (81.6)	7 (100)	7 (100)	14 (100)
Other	1 (4.8)	0	0	2 (6.9)	3 (9.7)	5 (5.7)	0	0	0
Metastases									
Yes	20 (95.2)	20 (100.0)	1 (14.3)	28 (96.6)	28 (90.3)	77 (88.5)	7 (100.0)	7 (100.0)	14 (100.0)
No	1 (4.8)	0	6 (85.7)	1 (3.4)	3 (9.7)	10 (11.5)	0	0	0
No. of prior systemic cancer therapy regimen	1.0 (0, 9)	2.0 (0, 4)	1.0 (0, 2)	1.0 (0, 9)	1.0 (0, 5)	1.0 (0, 9)	4.0 (2, 6)	4.0 (3, 7)	4.0 (2, 7)

Data are *n* (%) or median (range).

*dMMR* deficient mismatch repair, *ECOG PS* Eastern Cooperative Oncology Group performance status, *MSI-H* microsatellite instability-high, *Q3W* once every 3 weeks, *Q4W* once every 4 weeks, *Q6W* once every 6 weeks.

In part 2a, 14 patients with mCRC were treated with nofazinlimab 300 mg Q4W plus regorafenib 80 mg (*n* = 7) and 120 mg (*n* = 7). Overall, demographic characteristics in part 2a were similar between treatment groups. Median (range) age was 49.0 (37–70) years, and half of the patients were male. All patients were heavily pretreated, with 13/14 having had ≥3 prior anti-cancer therapy regimens. Ten (71.4%) patients, six in the regorafenib 80-mg group and four in the regorafenib 120-mg group, had known MSI/MMR status and none were MSI-H/dMMR.

### Safety

In part 1 phase 1a, all-grade and grade ≥3 AEs, irrespective of causality, were reported in 18 (85.7%) and 13 (61.9%) patients, respectively (Supplementary Table S1). Fifteen (71.4%) patients experienced TRAEs, most of which were grade 1 or 2 (Supplementary Table S2). TRAEs occurring in ≥10% of patients included fatigue, rash, pruritus, diarrhea, and nausea. Grade ≥3 TRAEs were reported in three (14.3%) patients (one grade 3 colitis, one grade 3 autoimmune hepatitis, and one patient with grade 4 lipase increased and grade 3 psoriasis). Only the latter two patients discontinued treatment due to these events. Two patients (9.5%) reported grade 1 infusion-related reactions (IRRs) and two (9.5%) deaths occurred due to AEs, which were deemed unrelated to nofazinlimab. No DLTs were observed in all four dose levels, and MTD was not reached; the 200 mg Q3W dosing regimen was selected as the RP2D.

In part 1 phase 1b, 85 (97.7%) patients experienced at least one treatment-emergent AE of any grade, with grade ≥3 AEs reported in 39 (44.8%) patients (Supplementary Table S3). TRAEs were reported in 49 (56.3%) patients, and grade 3 or 4 TRAEs were reported in five (5.7%) patients (Table 2). The most common (≥10% of patients) TRAEs were consistent with that seen in phase 1a (Table 2), and TRAEs led to treatment cessation in two (2.3%) patients. Thirty-seven (42.5%) patients reported serious AEs, including seven (8.0%) that were treatment-related. IRRs occurred in seven (8.0%) patients (all grade 1 or 2). One grade 5 AE (cardiac failure) occurred and was considered unrelated to nofazinlimab.

**Table 2:** Nofazinlimab-related adverse events reported in ≥10% patients or any grade ≥3 nofazinlimab-related adverse events in part 1 phase 1b

MedDRA Preferred Term	Arm 1 200 mg Q3W *n* = 20	Arm 2 200 mg Q3W *n* = 7	Arm 3 200 mg Q3W *n* = 29	Arm 4 400 mg Q6W *n* = 31	Total (*N* = 87)	
					Any grade	Grade ≥ 3
Number of patients with at least one event	11 (55.0)	5 (71.4)	13 (44.8)	20 (64.5)	49 (56.3)	5 (5.7)
Pruritus	3 (15.0)	1 (14.3)	3 (10.3)	6 (19.4)	13 (14.9)	0
Fatigue	3 (15.0)	1 (14.3)	4 (13.8)	4 (12.9)	12 (13.8)	0
Rash	2 (10.0)	0	4 (13.8)	3 (9.7)	9 (10.3)	0
Arthralgia	2 (10.0)	1 (14.3)	1 (3.4)	5 (16.1)	9 (10.3)	1 (1.1)
Myalgia	1 (5.0)	0	1 (3.4)	0	2 (2.3)	1 (1.1)
Type 1 diabetes mellitus	1 (5.0)	0	0	1 (3.2)	2 (2.3)	1 (1.1)
Dermatitis	0	0	0	1 (3.2)	1 (1.1)	1 (1.1)
Hyponatremia	1 (5.0)	0	0	0	1 (1.1)	1 (1.1)
Atrial flutter	1 (5.0)	0	0	0	1 (1.1)	1 (1.1)
Hepatitis	0	0	0	1 (3.2)	1 (1.1)	1 (1.1)

Data are *n* (%).

Only two (2.3%) patients in part 1 phase 1b discontinued nofazinlimab because of TRAEs: one patient experienced grade 3 hepatitis, and the other patient experienced grade 2 aspartate aminotransferase increased and grade 1 alanine aminotransferase increased.

*Q3W* once every 3 weeks, *Q6W* once every 6 weeks.

In part 2a, DLTs were reported in two patients: one in the nofazinlimab 300-mg Q4W/regorafenib 80-mg group (grade 3 colitis and maculo-papular rash; both were considered immune-related) and one in the nofazinlimab 300-mg Q4W/regorafenib 120-mg group (grade 2 pyrexia, unrelated to nofazinlimab but related to regorafenib, resulting in regorafenib interruption). All 14 patients experienced at least one AE, with grade 3/4 AEs, irrespective of causality, occurring in 12 (85.7%) patients (Supplementary Table S4). No grade 5 TEAEs were observed in part 2a. Nofazinlimab-related AEs were reported in nine (57.1%) patients, including three (21.4%) with grade 3/4 events (Table 3). The most common (≥20% of patients) nofazinlimab-related AEs included arthralgia and pyrexia (Table 3). Regorafenib-related AEs were reported in 11 (78.6%) patients, and seven (50.0%) patients experienced grade 3/4 events (Table 3). The most common (≥20% of patients) regorafenib-related AEs were palmar-plantar erythrodysesthesia syndrome, thrombocytopenia, maculo-papular rash, fatigue, and decreased appetite (Table 3). Eight (57.1%) patients reported serious AEs, including five that were deemed drug-related: grade 3 maculo-papular rash (*n* = 2, 14.3%), grade 4 seizure, grade 3 colitis, grade 3 aspartate aminotransferase increased, grade 3 transaminases increased, and grade 2 pyrexia (*n* = 1 each, 7.1%). Grade 1–3 IRRs occurred in two (14.3%) patients. One patient (14.3%) discontinued treatment with nofazinlimab due to a TRAE (grade 3 maculo-papular rash). TRAEs leading to dose interruption, dose reduction, and permanent discontinuation of regorafenib occurred in 78.6%, 14.3%, and 7.1% of patients, respectively. Any-grade TRAEs and grade ≥3 TRAEs in both dosing groups were similar.

**Table 3:** Treatment-related AEs reported in ≥10% patients and grade ≥3 treatment-related AEs in part 2a (*N* = 14).

MedDRA Preferred Term	Nofazinlimab treatment-related AE		Regorafenib treatment-related AE	
	Any grade	Grade ≥ 3	Any grade	Grade ≥ 3
Number of patients with at least one event	8 (57.1)	3 (21.4)	11 (78.6)	7 (50.0)
Pyrexia	3 (21.4)	1 (7.1)	1 (7.1)	0
Arthralgia	3 (21.4)	0	1 (7.1)	0
Fatigue	2 (14.3)	0	3 (21.4)	0
Rash	2 (14.3)	1 (7.1)	1 (7.1)	1 (7.1)
Maculo-papular rash^a^	2 (14.3)	2 (14.3)	3 (21.4)	2 (14.3)
Infusion-related reaction	2 (14.3)	0	2 (14.3)	0
Colitis	1 (7.1)	1 (7.1)	0	0
Lymphocyte count decreased	1 (7.1)	1 (7.1)	1 (7.1)	1 (7.1)
Transaminases increased	1 (7.1)	0	2 (14.3)	1 (7.1)
Seizure	1 (7.1)	1 (7.1)	0	0
Thrombocytopenia	1 (7.1)	1 (7.1)	4 (28.6)	1 (7.1)
Decreased appetite	1 (7.1)	0	3 (21.4)	0
Palmar-plantar erythrodysesthesia syndrome	0	0	4 (28.6)	1 (7.1)
Aspartate aminotransferase increased	0	0	1 (7.1)	1 (7.1)
Liver function test abnormal^a^	0	0	1 (7.1)	1 (7.1)
Neutropenia	0	0	2 (14.3)	1 (7.1)

Data are *n* (%).

*AE* adverse event.

^a^Two patients experienced treatment-related AEs leading to treatment discontinuation: one patient experienced grade 3 maculo-papular rash, which was related to nofazinlimab, and the other patient experienced grade 3 liver function test abnormal which was related to regorafenib.

### Efficacy

Tumor response and survival data are summarized in Table 4. In part 1 phase 1a, ORR was 14.3% (95% CI: 3.0%–36.3%) with DCR of 47.6% (95% CI: 25.7%–70.2%) among 21 patients who were response-evaluable, including one patient who achieved CR (stage IV Merkel cell carcinoma in the 3-mg/kg cohort) and two patients who achieved PR (one with MSI-H/dMMR CRC in the 200 mg fixed-dose group and one with stage IV basal cell carcinoma in the 10 mg/kg cohort). Median PFS was 2.8 months (95% CI: 1.7–6.0), and 33.3% and 14.3% of the patients were progression-free at 6 and 12 months, respectively.

**Table 4:** Tumor response and survival data (efficacy analysis set).

	Part 1 phase 1a *N* = 21	Part 1 phase 1b nofazinlimab monotherapy					Part 2a nofazinlimab combination (nofazinlimab 300 mg Q4W)		
		Arm 1	Arm 2	Arm 3	Arm 4				
		200 mg Q3W	200 mg Q3W	200 mg Q3W	400 mg Q6W	Total	Regorafenib 80 mg	Regorafenib 120 mg	Total
		*n* = 20	*n* = 7	*n* = 29	*n* = 31	*N* = 87	*n* = 7	*n* = 7	*N* = 14
ORR (CR + PR), *n* (%)	3 (14.3)	4 (20.0)	1 (14.3)	7 (24.1)	10 (32.3)	22 (25.3)	0	0	0
95% CI	3.0, 36.3	5.7, 43.7	0.4, 57.9	10.3, 43.5	16.7, 51.4	16.6, 35.7	NE, NE	NE, NE	NE, NE
Best overall response									
CR, *n* (%)	1 (4.8)	0	0	3 (10.3)	1 (3.2)	4 (4.6)	0	0	0
95% CI	0.1, 23.8	NE, NE	NE, NE	2.2, 27.4	0.1, 16.7	1.3, 11.4	NE, NE	NE, NE	NE, NE
PR, *n* (%)	2 (9.5)	4 (20.0)	1 (14.3)	4 (13.8)	9 (29.0)	18 (20.7)	0	0	0
95% CI	1.2, 30.4	5.7, 43.7	0.4, 57.9	3.9, 31.7	14.2, 48.0	12.7, 30.7	NE, NE	NE, NE	NE, NE
SD, *n* (%)	7 (33.0)	7 (35.0)	5 (71.4)	5 (17.2)	10 (32.3)	27 (31.0)	3 (42.9)	1 (14.3)	4 (28.6)
95% CI	14.6, 57.0	15.4, 59.2	29.0, 96.3	5.8, 35.8	16.7, 51.4	21.5, 41.9	9.9, 81.6	0.4, 57.9	8.4, 58.1
PD, *n*(%)	8 (38.1)	5 (25.0)	1 (14.3)	13 (44.8)	8 (25.8)	27 (31.0)	4 (57.1)	4 (57.1)	8 (57.1)
95% CI	18.1, 61.6	8.7, 49.1	0.4, 57.9	26.4, 64.3	11.9, 44.6	21.5, 41.9	18.4, 90.1	18.4, 90.1	28.9, 82.3
Not applicable, *n* (%)	3 (14.3)	4 (20.0)	0	4 (13.8)	3 (9.7)	11 (12.6)	0	2 (28.6)	2 (14.3)
DCR (CR + PR + SD), *n* (%)	10 (47.6)	11 (55.0)	6 (85.7)	12 (41.4)	20 (64.5)	49 (56.3)	3 (42.9)	1 (14.3)	4 (28.6)
95% CI	25.7, 70.2	31.5, 76.9	42.1, 99.6	23.5, 61.1	45.4, 80.8	45.3, 66.9	9.9, 81.6	0.4, 57.9	8.4, 58.1
DOR, months									
Median	12.8	NE	NE	NE	NE	NE	NE	NE	NE
95% CI	4.2, NE	9.0, NE	NE, NE	4.2, NE	4.1, NE	NE, NE	NE, NE	NE, NE	NE, NE
25th and 75th percentile	4.2, NE	9.0, NE	NE, NE	NE, NE	NE, NE	NE, NE	NE, NE	NE, NE	NE, NE
Range	4.2–24.0^a^	8.5^a^–16.8^a^	19.0^a^–19.0^a^	2.8^a^–16.6^a^	2.1^a^–13.8^a^	2.1^a^–19.0^a^	NE – NE	NE – NE	NE – NE
PFS, months									
Median	2.8	4.1	7.8	2.2	8.2	4.1	1.8	1.8	1.8
95% CI	1.7, 6.0	2.1, 10.6	1.3, 21.9	2.0, 7.9	2.1, NE	2.3, 8.1	1.7, NE	1.5, NE	1.7, 3.4
25th and 75th percentile	1.7, 6.1	2.1, 13.0	4.1, 21.9	1.9, 15.1	2.0, NE	2.0, 21.9	1.8, 5.5	1.7, 1.8	1.8, 3.4
Range	0.5–26.1^a^	1.1–23.5	1.3–21.9	0.9–23.2^a^	0.1–18.7^a^	0.1–23.5	1.7–13.6	0.0^a^–3.4	0.0^a^–13.6

*CI* confidence interval, *CR* complete response, *DCR* disease control rate, *DOR* duration of response, *NE* not estimable, *ORR* objective response rate, *PFS* progression-free survival, *PD* progressive disease, *PR* partial response, *Q3W* once every 3 weeks, *Q6W* once every 6 weeks, *SD* stable disease.

^a^Denotes minimum or maximum value from censored patients.

**Fig. 1:**
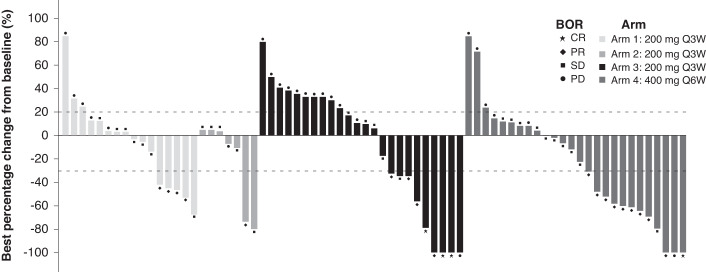
Best percentage change from baseline in tumor size of target lesions and best overall response in part 1 phase 1b. Selected tumor types in part 1 phase 1b evaluated included soft tissue sarcoma in arm 1, malignant pleural mesothelioma in arm 2, selected tumor types in basket arm 3 and arm 4. CR, complete response; PR, partial response; PD, progressive disease; Q3W, once every 3 weeks; Q6W, once every 6 weeks; SD, stable disease.

**Fig. 2:**
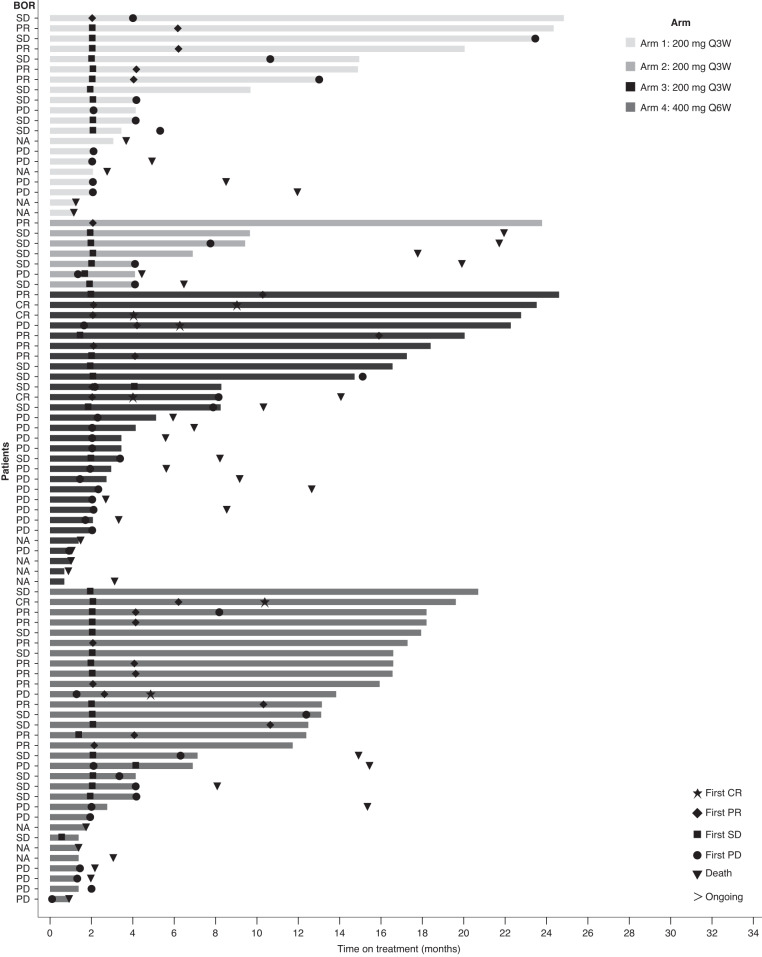
Treatment duration in part 1 phase 1b. Selected tumor types in part 1 phase 1b evaluated included soft tissue sarcoma in arm 1, malignant pleural mesothelioma in arm 2, selected tumor types in basket arm 3 and arm 4. BOR, best objective response; CR, complete response; NA, not applicable; PR, partial response; PD, progressive disease; Q3W, once every 3 weeks; Q6W, once every 6 weeks; SD, stable disease.

In part 1 phase 1b, ORR was 20.0% (95% CI: 5.7%–43.7%), 14.3% (95% CI: 0.4%–57.9%), 24.1% (95% CI: 10.3%–43.5%) and 32.3% (95% CI: 16.7%–51.4%) in arms 1–4, respectively. Three patients (10.3%) in arm 3 and one (3.2%) in arm 4 achieved a confirmed CR. Of the 11 patients with MSI-H/dMMR solid tumors, one patient with transitional cell carcinoma achieved a confirmed CR, one patient with endometrial adenocarcinoma and one patient with CRC achieved a confirmed PR. In nine patients with cutaneous squamous cell carcinoma, two achieved a confirmed CR, and one achieved a confirmed PR. In twelve patients with small-cell lung cancer, four patients achieved a confirmed PR. All patients with cutaneous squamous cell carcinoma and small-cell lung cancer had negative or unknown MSI status. Four patients with soft tissue sarcoma achieved a confirmed PR, including two patients with undifferentiated pleomorphic sarcoma, one patient with rhabdomyosarcoma, and one patient with angiosarcoma. The best percentage change from baseline in tumor size of target lesions in part 1 phase 1b is shown in Fig. 1. The overall DCR was 56.3% (95% CI: 45.3%–66.9%). Overall median DOR was not estimable. Median PFS was 4.1 months (95% CI: 2.3–8.1). Figure 2 shows a swimmer plot of treatment duration in part 1 phase 1b by arm.

In part 2a, no objective responses were observed. Three patients (42.9%) in the nofazinlimab 300-mg Q4W/regorafenib 80-mg group and one patient (14.3%) in the nofazinlimab 300-mg Q4W/regorafenib 120-mg group achieved the best overall response of stable disease. Overall median PFS was 1.8 months (95% CI: 1.7–3.4), and 6- and 12-month PFS rates were both 9.4%. A total of six patients (42.9%) died, all due to disease progression.

### Pharmacokinetics

Pharmacokinetic data after single and multiple dosing in part 1 phase 1a are summarized in Supplementary Table S5. The C_max_ and AUC_0-21d_ increased in a dose-proportional manner, from 1 mg/kg to 10 mg/kg Q3W, including at the 200-mg fixed dose. Following a single intravenous infusion, C_max_ (20.8–189 μg/mL) was achieved at the end of the infusion. The elimination half-life of nofazinlimab was approximately 12–14 days (291–335 h). After multiple intravenous infusions (cycle 4), the minimum concentrations were 1.94–98.9 µg/mL; clearance at steady state was 8.47–18.8 mL/h. The accumulation index for C_max_ and AUC were 1.24–1.70 and 1.56–1.98, respectively. Systemic exposure (AUC_0-21d,ss_ and C_min_) between 3 mg/kg and 200 mg/kg were similar. The PK properties of nofazinlimab is similar to that other marketed PD-1 of nivolumab, pembrolizumab and cemiplimab [19–21].

In part 1 phase 1b, the concentration–time profiles of arms 1–3 (nofazinlimab 200 mg Q3W) were comparable, whereas arm 4 (400 mg Q6W) had a higher C_max_ as expected. The mean C_trough_ was similar between the dosage of 200 mg Q3W and 400 mg Q6W.

In part 2a, no changes in nofazinlimab systemic exposure (C_max_ and AUC) were observed with increase in regorafenib dose. The PK exposure of regorafenib at steady state was similar with regorafenib monotherapy [22], indicating no significant PK interaction between the two agents.

### Biomarkers

In part 1 phase 1b, 73 patients had evaluable samples for PD-L1 expression, including 31 (42.5%) with TC% ≥1% and 68 (93.2%) with IC% ≥1%. The relationships between PD-L1 expression, tumor response, and PFS in part 1 phase 1b are summarized in Supplementary Table S6. ORR was 16.7% (95% CI: 7.0%–31.4%) versus 35.5% (95% CI: 19.2%–54.6%) for patients with TC < 1% versus TC ≥ 1% and was 0% versus 26.5% (95% CI: 16.5%–38.6%) for patients with IC < 1% versus IC ≥ 1%. Median PFS in part 1 phase 1b was 4.1 months (95% CI: 2.1–5.3) in patients with TC < 1% versus 8.2 months (95% CI: 2.1–23.5) in patients with TC ≥ 1% and 7.9 months (95% CI: 3.7–not estimable) in patients with IC < 1% versus 4.1 months (95% CI: 2.1–8.1) in patients with IC ≥ 1%.

## Discussion

In this first-in-human study of nofazinlimab, no DLTs were observed in part 1 phase 1a, and MTD was not reached at any of the escalating dose levels (1–10 mg/kg Q3W). TEAEs and TRAEs observed in patients did not reveal a clear dose-dependent increase in toxicity across doses. Preliminary anti-tumor activities were observed in 21 evaluable patients at dose of 3 mg/kg and above, including at 200 mg fixed dose. Receptor occupancy (RO) data from a separate phase 1 of nofazinlimab showed that the RO of PD-1 in peripheral T cells was close to saturation on C2D1 and remained saturated for at least 3 treatment cycles in 7 patients treated with 200 mg Q3W of nofazinlimab (the comprehensive data will be reported separately). Furthermore, the pharmacokinetic properties of nofazinlimab were comparable to those of other marketed anti-PD-1 mAbs, supporting the use of a flat dose and dosing interval of 3 weeks. In conclusion, the promising efficacy and safety of nofazinlimab from this phase 1a study, combined with a bridging study in China (separate manuscript in submission), led to selecting the recommended 200 mg Q3W dose (comparable to 3 mg/kg Q3W) as the RP2D. The ease of flat-dose administration further supports this choice.

The safety profile of nofazinlimab observed in this study was consistent with those reported for other anti-PD-L1/anti-PD-1 mAbs therapeutics [23]. Most TRAEs were grade 1 or 2 and were manageable. Only 9.5% (*n* = 2), 2.3% (*n* = 2), and 7.1% (*n* = 1) of the patients in phase 1a, phase 1b, and part 2a, respectively, discontinued nofazinlimab because of TRAEs.

The ORR in part 1 phase 1a and phase 1b in checkpoint inhibitor-naive patients with advanced solid tumors was 14.3% and 25.3%; the DCR was 47.6% and 56.3%, respectively. ORR was 20.0% and 14.3% in patients with soft tissue sarcoma (arm 1) and malignant pleural mesothelioma (arm 2), respectively. Specifically, ORR was 24.1% in arm 3 (200 mg Q3W) with three and four of 29 patients achieving CR and PR, respectively; while ORR was 32.3% in arm 4 (400 mg Q6W) with one and nine of 31 patients achieving CR and PR, respectively. Anti-tumor activity observed with nofazinlimab was comparable to that observed in studies of other single agent PD-1 inhibitors (e.g., nivolumab, pembrolizumab) in anti-PD-1/PD-L1-naïve populations with similar tumor types [24–28].

Because of the limited number of patients with suitable tissue for analysis, the relationship between PD-L1 expression and clinical responses was only assessed in the phase 1b portion. Although a formal statistical assessment was not conducted, a trend towards higher ORR and longer PFS was observed in patients with high PD-L1 expression (TC ≥ 1%) versus those with low PD-L1 expression (TC < 1%). A previous meta-analysis comprising of 41 clinical trials of PD-1/PD-L1 inhibitors with available PD-L1 biomarker data showed that tumor and tumor-infiltrating immune cell with PD-L1 overexpression was significantly associated with higher response rates to immune checkpoint inhibitors across a range of solid tumors. The significant association with higher response rates remained when PD-L1 expression was evaluated using different immunohistochemistry (IHC) assays [29]. Despite the widespread investigation in the clinical trials, the value of PD-L1 as a predictive biomarker varied among studies, in different tumor types and treatment settings. This may be due to inherent biological differences between tumor types, heterogenous expression of PD-L1 in the tumor, as well as the variability in IHC assays and cut-offs [30].

The toxicity profile of nofazinlimab combined with regorafenib was substantially heightened compared to nofazinlimab monotherapy and consistent with that reported previously for nivolumab administered in combination with regorafenib [17]. In total, 21.4% and 50.0% of patients experienced grade 3 or 4 AEs related to nofazinlimab or regorafeinib, respectively, although the majority were manageable with supportive measures and/or dose modifications. In particular, skin rash was a frequent TRAE (28.6%, grade 3/4 21.4%) in our study as was observed in the REGONIVO phase 1b study (42%) [17]. In REGONIVO, dose-escalation safety data showed that 160 mg regorafenib was not tolerable (three DLTs in three patients), while no DLTs were observed at 120- or 80-mg regorafenib combination with nivolumab [17]. In our study, one of seven patients presented with DLTs at each of the 80 mg and 120 mg regorafenib doses, and both doses combined with nofazinlimab 300 mg Q4W were determined as plausible RP2Ds.

In part 2a, there were no responders among patients with mCRC, and DCR was 28.6%. This result is consistent with other PD-1/PD-L1 inhibitor and regorafenib combination therapies in MSS mCRC, indicating that combined treatment with PD-1 inhibitor and regorafenib has limited benefit in MSS mCRC patients [31–34]. These results contrast with that of the REGONIVO study, which reported an ORR of 33.3% in the MSS mCRC cohort [17]. This may be partially explained by differences in ethnicity; all 25 patients with mCRC in REGONIVO were Japanese, while white patients comprised the majority in our study (78.6%–92.0%) and in others [31–34]. Furthermore, the RAS mutation rate in mCRC in REGONIVO was relatively low at 24%, but was 50% in our study (an additional 21% had unknown RAS mutational status) and 61%–71.2% of patients in the other aforementioned studies had RAS mutations [31–34]. Interestingly, both the Keynote 177 and Checkmate 142 studies of PD-1 mAbs in MSI-H/dMMR CRC showed numerically lower response rates in the KRAS/NRAS mutated subgroup, suggesting a possible negative correlation between RAS mutational status and immunotherapy response [35, 36]. The REGONIVO study noted a higher response rate in patients with lung metastases (8/16; 50.0%) compared with those with liver metastases (2/13; 15.4%) [17], with similar patterns observed in other studies [37]. In line with these findings, we observed no response among patients with liver metastasis. All four patients who achieved SD in our study had non-liver metastases, and two of them had lung metastases only. Preclinical studies have found that liver metastasis can induce a systematic immunosuppressive effect, such as lower CD8 + T-cell infiltration, thereby inhibiting antitumor activity [38]. Moreover, liver metastases also attract immunosuppressive macrophages that induce apoptosis of tumor antigen-specific T cells within the liver [39].

Nofazinlimab in combination with chemotherapy as first-line treatment in patients with extensive-stage small-cell lung cancer and in combination with lenvatinib as first-line treatment in patients with advanced hepatocellular carcinoma are currently being explored in a phase 1a/b study (NCT03809767) in mainland China. Additionally, a phase 3 randomized trial of nofazinlimab plus lenvatinib compared with placebo plus lenvatinib as first-line therapy in patients with advanced hepatocellular carcinoma is ongoing (NCT04194775) based on the promising anti-tumor activity observed in the phase 1 single-arm combination study (NCT03809767) [40, 41]. In addition, the combination of nofazinlimab and an anti-CTLA-4 antibody was explored in one phase 1a/1b study (NCT03523819); this combination demonstrated an encouraging anti-tumor activity and favorable safety profile in patients with MSI-H/dMMR tumors, anti-PD-(L)1-refractory melanoma and anti-PD-(L)1-refractory hepatocellular carcinoma [42, 43].

In conclusion, nofazinlimab was well tolerated and demonstrated preliminary anti-tumor activity in multiple tumor types at dose of 3 mg/kg and above. The fixed dose of 200 mg Q3W was determined as the RP2D based on all available data. An alternative dosing regimen of 400 mg Q6W showed comparable safety and efficacy, offering an additional and potentially more convenient option for patients and physicians, and could be further explored. Nofazinlimab 300 mg Q4W in combination with regorafenib at either 80 mg or 120 mg had a manageable safety profile, but no objective response was noted in a small cohort of heavily pretreated MSS mCRC patients.

### Supplementary information

Supplementary Materials

## Data Availability

The data are available for all study authors. The datasets used in the current analysis are available from the corresponding author upon reasonable request.
