# Supplementary material for: A first-in-human phase 1 study of nofazinlimab, an anti-PD-1 antibody, in advanced solid tumors and in combination with regorafenib in metastatic colorectal cancer
Source: Br J Cancer. 2023 Sep 20;129(10):1608–18. doi: 10.1038/s41416-023-02431-7 (PMC10646086; doi:10.1038/s41416-023-02431-7)
Supplement: Supplementary file 1 — Supplementary Materials [file 41416_2023_2431_MOESM1_ESM.docx]

# Supplementary materials

Supplementary Table S1. Adverse events reported in ≥10% of patients and grade ≥3 adverse events in part 1 phase 1a

Supplementary Table S2. Nofazinlimab-related adverse events and grade ≥3 nofazinlimab-related adverse events in part 1 phase 1a

Supplementary Table S3. Adverse events reported in ≥10% patients and grade ≥3 adverse events reported in ≥2 patients in part 1 phase 1b

Supplementary Table S4. Adverse events reported in ≥10% patients and grade ≥3 adverse events in part 2a

Supplementary Table S5. Summary statistics for serum pharmacokinetic parameters after single and multiple dosing in part 1 phase 1a

Supplementary Table S6. Tumor response by PD-L1 expression level in patients treated with nofazinlimab in part 1 phase 1b

Supplementary Figure S1. Study design

Supplementary Methods

Additional details of the study methods

Definition of dose-limiting toxicities (DLT)

Inclusion criteria

Exclusion criteria

**Supplementary Table S1.** Adverse events reported in ≥10% of patients and grade ≥3 adverse events in phase 1a

| **Preferred Term** | **Nofazinlimab**  **1 mg/kg**  ***n* = 3** | **Nofazinlimab**  **3 mg/kg**  ***n* = 5** | **Nofazinlimab 200 mg**  ***n* = 8** | **Nofazinlimab**  **10 mg/kg**  ***n* = 5** | **Total (*N* = 21)** | |
| --- | --- | --- | --- | --- | --- | --- |
|  |  |  |  |  | **Any grade** | **Grade ≥3** |
| Number of patients with at least one event | 3 (100.0) | 4 (80.0) | 6 (75.0) | 5 (100.0) | 18 (85.7) | 13 (61.9) |
| Fatigue | 3 (100.0) | 2 (40.0) | 2 (25.0) | 2 (40.0) | 9 (42.9) | 0 |
| Rash | 1 (33.3) | 1 (20.0) | 2 (25.0) | 2 (40.0) | 6 (28.6) | 0 |
| Diarrhea | 0 | 2 (40.0) | 2 (25.0) | 1 (20.0) | 5 (23.8) | 0 |
| Nausea | 2 (66.7) | 1 (20.0) | 2 (25.0) | 0 | 5 (23.8) | 0 |
| Vomiting | 1 (33.3) | 1 (20.0) | 1 (12.5) | 1 (20.0) | 4 (19.0) | 0 |
| Pyrexia | 0 | 2 (40.0) | 1 (12.5) | 1 (20.0) | 4 (19.0) | 2 (9.5) |
| Pruritus | 1 (33.3) | 0 | 1 (12.5) | 2 (40.0) | 4 (19.0) | 0 |
| Decreased appetite | 1 (33.3) | 1 (20.0) | 1 (12.5) | 1 (20.0) | 4 (19.0) | 0 |
| Anemia | 1 (33.3) | 2 (40.0) | 0 | 1 (20.0) | 4 (19.0) | 3 (14.3) |
| Abdominal pain | 0 | 1 (20.0) | 2 (25.0) | 0 | 3 (14.3) | 1 (4.8) |
| Oedema peripheral | 1 (33.3) | 1 (20.0) | 1 (12.5) | 0 | 3 (14.3) | 0 |
| Cough | 1 (33.3) | 0 | 2 (25.0) | 0 | 3 (14.3) | 0 |
| Dizziness | 1 (33.3) | 1 (20.0) | 1 (12.5) | 0 | 3 (14.3) | 0 |
| Headache | 0 | 1 (20.0) | 1 (12.5) | 1 (20.0) | 3 (14.3) | 0 |
| Dehydration | 0 | 1 (20.0) | 1 (12.5) | 1 (20.0) | 3 (14.3) | 1 (4.8) |
| Hot flush | 1 (33.3) | 1 (20.0) | 1 (12.5) | 0 | 3 (14.3) | 0 |
| General physical health deterioration | 0 | 1 (20.0) | 0 | 1 (20.0) | 2 (9.5) | 1 (4.8) |
| Dyspnea | 0 | 2 (40.0) | 0 | 0 | 2 (9.5) | 1 (4.8) |
| Pulmonary embolism | 1 (33.3) | 0 | 1 (12.5) | 0 | 2 (9.5) | 2 (9.5) |
| Hypercalcemia | 1 (33.3) | 0 | 0 | 1 (20.0) | 2 (9.5) | 1 (4.8) |
| Lipase increased | 0 | 0 | 2 (25.0) | 0 | 2 (9.5) | 1 (4.8) |
| Colitis | 0 | 1 (20.0) | 0 | 0 | 1 (4.8) | 1 (4.8) |
| Enterocutaneous fistula | 0 | 0 | 1 (12.5) | 0 | 1 (4.8) | 1 (4.8) |
| Multiple organ dysfunction syndrome | 0 | 0 | 1 (12.5) | 0 | 1 (4.8) | 1 (4.8) |
| Puncture site pain | 0 | 0 | 1 (12.5) | 0 | 1 (4.8) | 1 (4.8) |
| Pleural effusion | 0 | 1 (20.0) | 0 | 0 | 1 (4.8) | 1 (4.8) |
| Psoriasis | 0 | 0 | 1 (12.5) | 0 | 1 (4.8) | 1 (4.8) |
| Syncope | 0 | 0 | 0 | 1 (20.0) | 1 (4.8) | 1 (4.8) |
| Hypoalbuminemia | 1 (33.3) | 0 | 0 | 0 | 1 (4.8) | 1 (4.8) |
| Abdominal sepsis | 0 | 0 | 1 (12.5) | 0 | 1 (4.8) | 1 (4.8) |
| Gastroenteritis viral | 0 | 0 | 1 (12.5) | 0 | 1 (4.8) | 1 (4.8) |
| Sepsis | 0 | 0 | 1 (12.5) | 0 | 1 (4.8) | 1 (4.8) |
| Blood lactate dehydrogenase increased | 0 | 0 | 0 | 1 (20.0) | 1 (4.8) | 1 (4.8) |
| Gamma-glutamyltransferase increased | 0 | 0 | 0 | 1 (20.0) | 1 (4.8) | 1 (4.8) |
| Myelosuppression | 0 | 1 (20.0) | 0 | 0 | 1 (4.8) | 1 (4.8) |
| Right ventricular failure | 0 | 0 | 1 (12.5) | 0 | 1 (4.8) | 1 (4.8) |
| Autoimmune hepatitis | 0 | 0 | 1 (12.5) | 0 | 1 (4.8) | 1 (4.8) |

Data are *n* (%).

**Supplementary Table S2.** Nofazinlimab-related adverse events and grade ≥3 nofazinlimab-related adverse events in phase 1a

| **Preferred Term** | **Nofazinlimab  1 mg/kg   *n* = 3** | **Nofazinlimab  3 mg/kg   *n* = 5** | **Nofazinlimab  200 mg   *n* = 8** | **Nofazinlimab 10 mg/kg  *n* = 5** | **Total (*N* = 21)** | |
| --- | --- | --- | --- | --- | --- | --- |
|  |  |  |  |  | **Any grade** | **Grade ≥3** |
| Number of patients with at least one event | 3 (100.0) | 4 (80.0) | 4 (50.0) | 4 (80.0) | 15 (71.4) | 3 (14.3) |
| Fatigue | 1 (33.3) | 2 (40.0) | 2 (25.0) | 1 (20.0) | 6 (28.6) | 0 |
| Rash | 1 (33.3) | 1 (20.0) | 1 (12.5) | 2 (40.0) | 5 (23.8) | 0 |
| Pruritus | 1 (33.3) | 0 | 1 (12.5) | 2 (40.0) | 4 (19.0) | 0 |
| Diarrhea | 0 | 2 (40.0) | 0 | 1 (20.0) | 3 (14.3) | 0 |
| Nausea | 2 (66.7) | 0 | 1 (12.5) | 0 | 3 (14.3) | 0 |
| Dizziness | 1 (33.3) | 0 | 1 (12.5) | 0 | 2 (9.5) | 0 |
| Hyperthyroidism | 0 | 1 (20.0) | 0 | 1 (20.0) | 2 (9.5) | 0 |
| Hyperhidrosis | 1 (33.3) | 0 | 0 | 0 | 1 (4.8) | 0 |
| Psoriasis | 0 | 0 | 1 (12.5) | 0 | 1 (4.8) | 1 (4.8) |
| Skin disorder | 0 | 0 | 0 | 1 (20.0) | 1 (4.8) | 0 |
| Colitis | 0 | 1 (20.0) | 0 | 0 | 1 (4.8) | 1 (4.8) |
| Chills | 0 | 0 | 0 | 1 (20.0) | 1 (4.8) | 0 |
| Pyrexia | 0 | 0 | 0 | 1 (20.0) | 1 (4.8) | 0 |
| Lethargy | 0 | 0 | 1 (12.5) | 0 | 1 (4.8) | 0 |
| Amylase increased | 0 | 0 | 1 (12.5) | 0 | 1 (4.8) | 0 |
| Blood thyroid stimulating hormone decreased | 0 | 0 | 1 (12.5) | 0 | 1 (4.8) | 0 |
| Lipase increased | 0 | 0 | 1 (12.5) | 0 | 1 (4.8) | 1 (4.8) |
| Thrombocytopenia | 0 | 0 | 1 (12.5) | 0 | 1 (4.8) | 0 |
| Autoimmune hepatitis | 0 | 0 | 1 (12.5) | 0 | 1 (4.8) | 1 (4.8) |
| Decreased appetite | 1 (33.3) | 0 | 0 | 0 | 1 (4.8) | 0 |
| Pneumonitis | 0 | 0 | 0 | 1 (20.0) | 1 (4.8) | 0 |

**Supplementary Table S3.** Adverse events reported in ≥10% patients and grade ≥3 adverse events reported in ≥2 patients in phase 1b

| **Preferred Term** | **Arm 1 200 mg Q3W *n* = 20** | **Arm 2 200 mg Q3W *n* = 7** | **Arm 3 200 mg Q3W *n* = 29** | **Arm 4 400 mg Q6W *n* = 31** | **Total (*N* = 87)** | |
| --- | --- | --- | --- | --- | --- | --- |
|  |  |  |  |  | **Any grade** | **Grade ≥3** |
| Number of patients with at least one event | 19 (95.0) | 7 (100.0) | 28 (96.6) | 31 (100.0) | 85 (97.7) | 39 (44.8) |
| Fatigue | 8 (40.0) | 3 (42.9) | 12 (41.4) | 12 (38.7) | 35 (40.2) | 2 (2.3) |
| Arthralgia | 7 (35.0) | 1 (14.3) | 4 (13.8) | 7 (22.6) | 19 (21.8) | 1 (1.1) |
| Diarrhea | 4 (20.0) | 2 (28.6) | 5 (17.2) | 5 (16.1) | 16 (18.4) | 0 |
| Nausea | 4 (20.0) | 1 (14.3) | 6 (20.7) | 5 (16.1) | 16 (18.4) | 0 |
| Constipation | 2 (10.0) | 2 (28.6) | 4 (13.8) | 7 (22.6) | 15 (17.2) | 0 |
| Rash | 4 (20.0) | 1 (14.3) | 5 (17.2) | 5 (16.1) | 15 (17.2) | 1 (1.1) |
| Cough | 5 (25.0) | 2 (28.6) | 4 (13.8) | 4 (12.9) | 15 (17.2) | 0 |
| Pruritus | 3 (15.0) | 1 (14.3) | 4 (13.8) | 6 (19.4) | 14 (16.1) | 0 |
| Dyspnea | 3 (15.0) | 2 (28.6) | 3 (10.3) | 5 (16.1) | 13 (14.9) | 1 (1.1) |
| Abdominal pain | 3 (15.0) | 0 | 7 (24.1) | 2 (6.5) | 12 (13.8) | 2 (2.3) |
| Urinary tract infection | 3 (15.0) | 1 (14.3) | 3 (10.3) | 5 (16.1) | 12 (13.8) | 2 (2.3) |
| Headache | 4 (20.0) | 1 (14.3) | 3 (10.3) | 3 (9.7) | 11 (12.6) | 0 |
| Influenza-like illness | 4 (20.0) | 0 | 4 (13.8) | 2 (6.5) | 10 (11.5) | 0 |
| Pyrexia | 2 (10.0) | 3 (42.9) | 1 (3.4) | 4 (12.9) | 10 (11.5) | 0 |
| Dry mouth | 2 (10.0) | 2 (28.6) | 1 (3.4) | 5 (16.1) | 10 (11.5) | 0 |
| Vomiting | 2 (10.0) | 1 (14.3) | 4 (13.8) | 3 (9.7) | 10 (11.5) | 1 (1.1) |
| Decreased appetite | 3 (15.0) | 1 (14.3) | 2 (6.9) | 4 (12.9) | 10 (11.5) | 0 |
| Hypothyroidism | 3 (15.0) | 0 | 3 (10.3) | 3 (9.7) | 9 (10.3) | 0 |
| Anemia | 0 | 0 | 5 (17.2) | 3 (9.7) | 8 (9.2) | 4 (4.6) |
| Pneumonia | 0 | 1 (14.3) | 3 (10.3) | 0 | 4 (4.6) | 3 (3.4) |
| Ascites | 0 | 0 | 3 (10.3) | 0 | 3 (3.4) | 2 (2.3) |
| Obstruction gastric | 1 (5.0) | 0 | 0 | 1 (3.2) | 2 (2.3) | 2 (2.3) |
| Small intestinal obstruction | 0 | 0 | 2 (6.9) | 0 | 2 (2.3) | 2 (2.3) |
| Pulmonary embolism | 0 | 0 | 1 (3.4) | 1 (3.2) | 2 (2.3) | 2 (2.3) |
| Hyponatremia | 1 (5.0) | 0 | 0 | 1 (3.2) | 2 (2.3) | 2 (2.3) |

Data are *n* (%).

Q3W, once every 3 weeks; Q6W, once every 6 weeks.

**Supplementary Table S4.** Adverse events reported in ≥10% patients and grade ≥3 adverse events in part 2a

| **Preferred Term** | **Regorafenib 80 mg + nofazinlimab 300 mg Q4W *n* = 7** | **Regorafenib 120 mg + nofazinlimab 300 mg Q4W *n* = 7** | **Total (*N* = 14)** | |
| --- | --- | --- | --- | --- |
|  |  |  | **Any grade** | **Grade ≥3** |
| Number of patients with at least one event | 7 (100.0) | 7 (100.0) | 14 (100.0) | 12 (85.7) |
| Fatigue | 2 (28.6) | 4 (57.1) | 6 (42.9) | 0 |
| Maculo-papular rash | 2 (28.6) | 3 (42.9) | 5 (35.7) | 4 (28.6) |
| Decreased appetite | 3 (42.9) | 2 (28.6) | 5 (35.7) | 0 |
| Palmar-plantar erythrodysesthesia syndrome | 1 (14.3) | 3 (42.9) | 4 (28.6) | 1 (7.1) |
| Thrombocytopenia | 2 (28.6) | 2 (28.6) | 4 (28.6) | 1 (7.1) |
| Pyrexia | 1 (14.3) | 2 (28.6) | 3 (21.4) | 1 (7.1) |
| Arthralgia | 0 | 3 (42.9) | 3 (21.4) | 0 |
| Back pain | 1 (14.3) | 2 (28.6) | 3 (21.4) | 0 |
| Headache | 1 (14.3) | 2 (28.6) | 3 (21.4) | 0 |
| Abdominal pain | 0 | 2 (28.6) | 2 (14.3) | 2 (14.3) |
| Diarrhea | 1 (14.3) | 1 (14.3) | 2 (14.3) | 0 |
| Nausea | 1 (14.3) | 1 (14.3) | 2 (14.3) | 0 |
| Vomiting | 0 | 2 (28.6) | 2 (14.3) | 0 |
| Rash | 0 | 2 (28.6) | 2 (14.3) | 1 (7.1) |
| Myalgia | 1 (14.3) | 1 (14.3) | 2 (14.3) | 0 |
| Neutropenia | 1 (14.3) | 1 (14.3) | 2 (14.3) | 1 (7.1) |
| Transaminases increased | 1 (14.3) | 1 (14.3) | 2 (14.3) | 1 (7.1) |
| Cough | 1 (14.3) | 1 (14.3) | 2 (14.3) | 0 |
| Pneumonia | 0 | 2 (28.6) | 2 (14.3) | 1 (7.1) |
| Infusion-related reaction | 0 | 2 (28.6) | 2 (14.3) | 0 |
| Colitis | 1 (14.3) | 0 | 1 (7.1) | 1 (7.1) |
| Intestinal obstruction | 0 | 1 (14.3) | 1 (7.1) | 1 (7.1) |
| Anemia | 1 (14.3) | 0 | 1 (7.1) | 1 (7.1) |
| Aspartate aminotransferase increased | 0 | 1 (14.3) | 1 (7.1) | 1 (7.1) |
| Liver function test abnormal | 1 (14.3) | 0 | 1 (7.1) | 1 (7.1) |
| Lymphocyte count decreased | 0 | 1 (14.3) | 1 (7.1) | 1 (7.1) |
| Radiculopathy | 1 (14.3) | 0 | 1 (7.1) | 1 (7.1) |
| Dyspnea | 0 | 1 (14.3) | 1 (7.1) | 1 (7.1) |
| Biliary obstruction | 0 | 1 (14.3) | 1 (7.1) | 1 (7.1) |
| Cholangitis | 0 | 1 (14.3) | 1 (7.1) | 1 (7.1) |
| Hepatitis cholestatic | 0 | 1 (14.3) | 1 (7.1) | 1 (7.1) |
| Biliary tract infection | 0 | 1 (14.3) | 1 (7.1) | 1 (7.1) |
| Seizure | 0 | 1 (14.3) | 1 (7.1) | 1 (7.1) |
| Embolism | 1 (14.3) | 0 | 1 (7.1) | 1 (7.1) |

Data are *n* (%).

Q4W, once every 4 weeks.

**Supplementary Table S5.** Summary statistics for serum pharmacokinetic parameters after single and multiple dosing in part 1 phase 1a

| **Parameter (unit)** | **1 mg/kg**  ***n* = 3** | | **3 mg/kg**  ***n* = 5** | | **200 mg**  ***n* = 8** | | **10 mg/kg**  ***n* = 5** | |
| --- | --- | --- | --- | --- | --- | --- | --- | --- |
|  | ***n*** | **Geometric mean (Geometric CV%)** | ***n*** | **Geometric mean (Geometric CV%)** | ***n*** | **Geometric mean (Geometric CV %)** | ***n*** | **Geometric mean (Geometric CV%)** |
| **Cycle 1** |  |  |  |  |  |  |  |  |
| AUC_0-∞_ (h•µg/mL) | 1 | 2180 | 1 | 14700 | 1 | 12800 |  |  |
| AUC_0-21d_ (h•µg/mL) | 3 | 3360 (54.14) | 5 | 12000 (13.31) | 8 | 9860 (36.89) | 5 | 39800 (26.98) |
| C_max_ (µg/mL) | 3 | 20.8 (25.35) | 5 | 58.4 (13.86) | 8 | 51.1 (31.88) | 5 | 189 (15.98) |
| T_max_ (h)^a^ | 3 | 3.00 (2.23, 3.17) | 5 | 3.00 (2.08, 3.13) | 8 | 4.81 (2.00, 7.57) | 5 | 3.12 (2.05, 25.5) |
| t_1/2_ (h) | 3 | 323 (57.96) | 4 | 327 (42.21) | 8 | 335 (61.79) | 4 | 291(54.08) |
| CL (mL/h) | 1 | 28.0 | 1 | 13.6 | 1 | 15.6 |  |  |
| Vz (mL) | 1 | 7280 | 1 | 3630 | 1 | 5120 |  |  |
| **Cycle 4** |  |  |  |  |  |  |  |  |
| AUC_0-21d,ss_ (h•µg/mL) | 2 | 4310 (158.15) | 4 | 23600 (10.72) | 5 | 15800 (46.25) | 2 | 87800 (32.47) |
| C_max,ss_ (µg/mL) | 2 | 23.1 (58.72) | 4 | 87.9 (20.53) | 5 | 77.6 (39.67) | 2 | 314 (10.83) |
| T_max_ (h)^a^ | 2 | 3.15 (3.13, 3.17) | 4 | 2.62 (2.05, 25.5) | 5 | 1.98 (0.97, 3.58) | 2 | 5.11 (3.05, 7.17) |
| t_1/2_ (h) | 2 | 195 (118.13) | 3 | 338 (14.53) | 5 | 386 (39.2) | 2 | 505 (19.53) |
| CL_ss_ (mL/h) | 2 | 18.8 (82.72) | 4 | 8.47 (22.60) | 5 | 12.6 (46.25) | 2 | 9.13 (10.68) |
| V_ss_ (mL) | 1 | 4610 |  |  |  |  |  |  |
| C_min,ss_ (µg/mL) | 2 | 1.94 (1381.73) | 4 | 27.0 (14.99) | 5 | 16.6 (54.05) | 2 | 98.9 (15.14) |
| Racc_(AUC)_ | 2 | 1.56 (64.47) | 4 | 1.98 (12.29) | 5 | 1.62 (26.74) | 2 | 1.72 (6.07) |
| Racc_(Cmax)_ | 2 | 1.24 (32.61) | 4 | 1.53 (15.42) | 5 | 1.70 (35.96) | 2 | 1.53 (9.77) |

^a^T_max_ data are median (min, max).

AUC, area under the concentration–time curve; AUC_0-21d_, area under the concentration–time curve from the time of dosing to day 21; AUC_0-∞_, area under the concentration–time curve to infinite time; CL, clearance; CL_ss_, CL, clearance at steady state; n, number of patients; N, number of patients in the analysis set.

Racc_(AUC)_, accumulation index (based on AUC), calculated as AUC_0-21d_ at cycle 4/ AUC_0-21d_ at cycle 1;

Racc_(Cmax)_, accumulation ratio (based on C_max_), calculated as C_max_ at cycle 4/ C_max_ at cycle 1; T_max_, time to maximum observed serum concentration; t_1/2_, half-life; V_ss,_ volume of distribution at steady state; Vz, volume of distribution during the terminal phase.

**Supplementary Table S6.** Tumor response by PD-L1 expression level in patients treated with nofazinlimab in part 1 phase 1b

| **Response** | TC% <1%  ***n* = 42** | TC% ≥1%  ***n* = 31** | IC% <1%  ***n* = 5** | IC% ≥1%  ***n* = 68** |
| --- | --- | --- | --- | --- |
| ORR (CR+PR), *n* (%) | 7 (16.7) | 11 (35.5) | 0 | 18 (26.5) |
| 95% CI | 7.0, 31.4 | 19.2, 54.6 | NE, NE | 16.5, 38.6 |
|  |  |  |  |  |
| **Best overall response** |  |  |  |  |
| CR, *n* (%) | 2 (4.8) | 1 (3.2) | 0 | 3 (4.4) |
| 95% CI | 0.6, 16.2 | 0.1, 16.7 | NE, NE | 0.9, 12.4 |
| PR, *n* (%) | 5 (11.9) | 10 (32.3) | 0 | 15 (22.1) |
| 95% CI | 4.0, 25.6 | 16.7, 51.4 | NE, NE | 12.9, 33.8 |
| SD, *n* (%) | 16 (38.1) | 8 (25.8) | 4 (80.0) | 20 (29.4) |
| 95% CI | 23.6, 54.4 | 11.9, 44.6 | 28.4, 99.5 | 19.0, 41.7 |
| PD, *n* (%) | 12 (28.6) | 11 (35.5) | 0 | 23 (33.8) |
| 95% CI | 15.7, 44.6 | 19.2, 54.6 | NE, NE | 22.8, 46.3 |
| Not applicable, *n* (%) | 7 (16.7%) | 1 (3.2) | 1 (20.0) | 7 (10.3) |
|  |  |  |  |  |
| DCR (CR+PR+SD), *n* (%) | 23 (54.8%) | 19 (61.3) | 4 (80.0) | 38 (55.9) |
| 95% CI | 38.7, 70.2 | 42.2, 78.2 | 28.4, 99.5 | 43.3, 67.9 |
|  |  |  |  |  |
| **PFS Statistic** |  |  |  |  |
| Patients with event, *n* (%) | 32 (76.2) | 20 (64.5) | 3 (60.0) | 49 (72.1) |
| Death | 7 | 2 | 1 | 8 |
| Progressive disease | 25 | 18 | 2 | 41 |
| Patients censored, *n* (%) | 10 (23.8) | 11 (35.5) | 2 (40.0) | 19 (27.9) |
| PFS (months) |  |  |  |  |
| Median | 4.1 | 8.2 | 7.9 | 4.1 |
| 95% CI | 2.1, 5.3 | 2.1, 23.5 | 3.7, NE | 2.1, 8.1 |
| 25^th^ and 75^th^ percentiles | 2.0, 15.1 | 2.1, 23.5 | 4.1, NE | 2.0, 23.5 |
| Range | 0.9–20.6^+^ | 0.9–23.5 | 3.7–8.2^+^ | 0.9–23.5 |
| PFS rate (%) |  |  |  |  |
| 3 months | 57.1 | 64.5 | 100.0 | 57.4 |
| 95% CI | 40.9, 70.4 | 45.2, 78.5 | 100.0, 100.0 | 44.8, 68.1 |
| 6 months | 35.7 | 54.8 | 60.0 | 42.6 |
| 95% CI | 21.7, 49.9 | 36.0, 70.3 | 12.6, 88.2 | 30.8, 54.0 |
| 9 months | 28.6 | 48.0 |  | 36.8 |
| 95% CI | 16.0, 42.5 | 29.7, 64.1 |  | 25.5, 48.1 |
| 12 months | 28.6 | 44.3 |  | 35.2 |
| 95% CI | 16.0, 42.5 | 26.4, 60.8 |  | 24.1, 46.5 |
| 15 months | 25.7 | 39.9 |  | 31.9 |
| 95% CI | 13.6, 39.6 | 22.3, 56.9 |  | 21.1, 43.1 |
| 18 months | 22.0 | 34.9 |  | 27.4 |
| 95% CI | 10.5, 36.2 | 17.8, 52.6 |  | 16.9, 38.9 |
| 21 months |  | 34.9 |  | 27.4 |
| 95% CI |  | 17.8, 52.6 |  | 16.9, 38.9 |

CI, confidence interval; CR, complete response; DCR, disease control rate; DOR, duration of response; IC%, percentage of tumor-infiltrating immune cells with positive staining of PD-L1; NE, not estimable; ORR, objective response rate; PFS, progression-free survival; PD, progressive disease; PD-L1, programmed death ligand-1; PR, partial response; Q3W, once every 3 weeks; Q6W, once every 6 weeks; SD, stable disease; TC%, percentage of tumor cells.

**Supplementary Figure S1.** **Study Design**


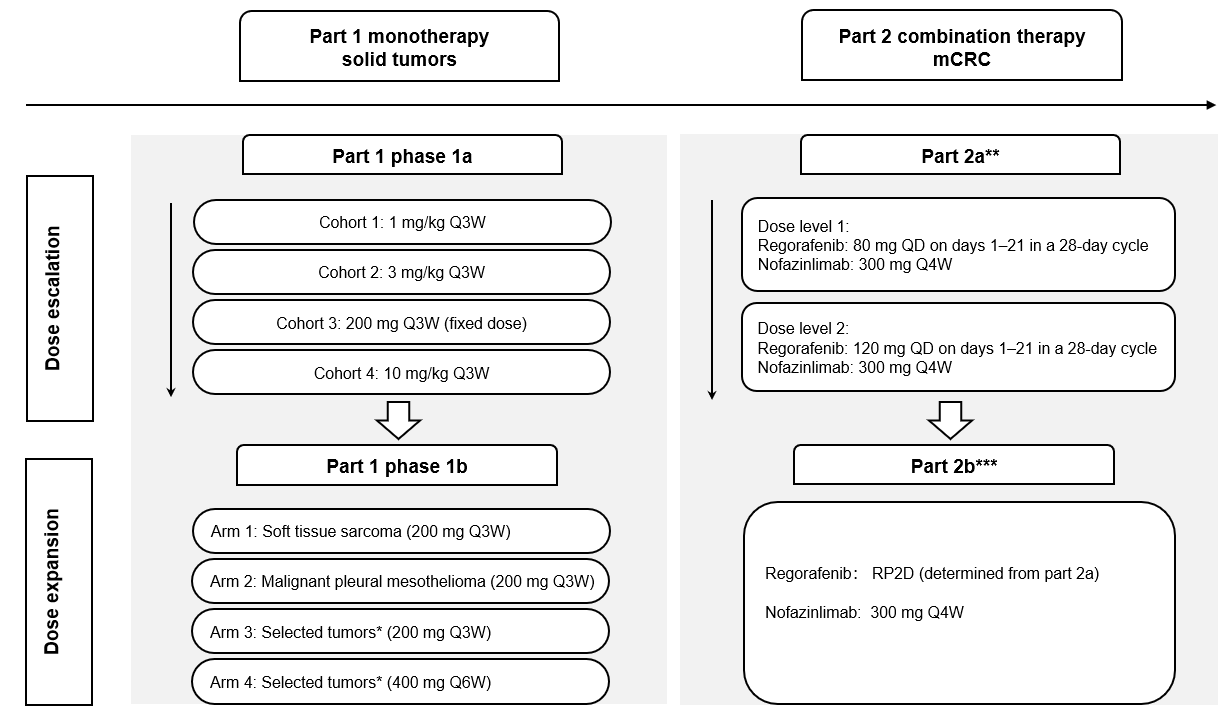


* Selected tumors included bladder cancer, Merkel-cell carcinoma, gastric cancer, esophageal carcinoma, small-cell lung cancer, large-cell lung cancer, head and neck squamous cell carcinoma or cutaneous squamous cell carcinoma. Any solid tumors with microsatellite instability-high or deficient mismatch repair, or other tumor types after discussion with the medical monitor and in consultation with the Sponsor.

** In part 2a, nofazinlimab was administered intravenously at 300 mg Q4W combined with regorafenib administered orally at 80 mg (dose level 1) and 120 mg (dose level 2) once daily (QD) for the first 21 days of each 28-day cycle. If these dose regimens were considered intolerable, other dosing schedules planned would proceed.

*** Part 2b was planned but did not proceed.

QD, once daily; Q3W, once every 3 weeks; Q4W, once every 4 weeks; Q6W, once every 6 weeks; RP2D, recommended phase 2 dose.

**Supplementary Methods**

**Additional details of the study methods**

Disease assessment by radiographic imaging (computed tomography or magnetic resonance imaging) was performed and recorded at screening within 28 days before enrollment and approximately every 9 weeks (± 5 days) in the first year for part 1 and every 8 weeks (± 5 days) within 1 year for part 2, and every 12 weeks (± 5 days) for both parts 1 and 2 thereafter.

In part 1 phase 1a, approximately 12–24 patients would be required to establish the maximum tolerated dose (MTD) and recommended phase 2 dose (RP2D) of nofazinlimab when administered as a single agent. In part 1 phase 1b, approximately 60–90 patients would be required to evaluate the preliminary efficacy of nofazinlimab when administered as a single agent. In part 2a and 2b, approximately 30–40 patients were required to establish the MTD and RP2D of regorafenib when administered in combination with nofazinlimab and evaluate the preliminary efficacy of nofazinlimab when administered in combination with regorafenib at the RP2D detected from part 2a.

Nofazinlimab administered intravenously at 300 mg Q4W were evaluated in combination with regorafenib administered orally at 2 dose levels: 80 mg and 120 mg, QD (dose levels 1–2, respectively) for the first 21 days of each 28-day cycle. If these dose regimens were considered intolerable, two alternative dosing schedules of nofazinlimab administered intravenously at 300 mg every 4 weeks (Q4W) in combination with regorafenib would be evaluated upon approval by the Safety Monitoring Committee: (i) 80 mg and 120 mg daily (QD), taken orally every other week (1-week on/1-week off treatment) in each 28-day cycle (dose levels 1A and 2A, respectively), or (ii) 80 mg and 120 mg every other day, taken orally continuously in each 28-day cycle (dose levels 1B and 2B, respectively). If dose level 1A or dose level 1B was considered tolerable, the Safety Monitoring Committee may decide either of the options: 1) to escalate to the dose of regorafenib to dose level 2A or 2B, respectively; 2) or to re-evaluate dose level 1 in three additional patients (under the premise of no more than two patients being observed DLTs out of six patients at the first evaluation of dose level 1; if no DLT is observed in these additional three patients, dose level 1 will be declared RP2D and no further escalation of dose level 2 would be assessed; re-evaluation of dose level 1 would be denied when at least two patients experience DLTs among less than six patients in cycle 1 at the first evaluation).

Efficacy (objective response rate, disease control rate, duration of response, progression-free survival, overall survival) was evaluated using the efficacy analysis set, defined as all patients with measurable baseline disease who received at least one dose of the study drug. Safety was evaluated using the safety analysis set, defined as all patients who received at least one dose of the study drug. The pharmacokinetic analysis was evaluated using the pharmacokinetic analysis set, defined as all patients with at least one measurable concentration after baseline who received at least one dose of the study drug. The immunogenicity analysis set consisted of all patients with at least one drug administration and at least one reportable antidrug antibody result. The safety analysis set with available biomarker results was used for the biomarker analysis. The MTD was evaluated using the dose-determining set, defined as all patients from the safety set who, in cycle 1, either met the minimum exposure criterion and were considered by the Safety Monitoring Committee to have sufficient safety evaluations or discontinued due to dose-limiting toxicity.**Definition of dose-limiting toxicities (DLT)**

DLT for part 1 phase 1a

All toxicities were graded according to the National Cancer Institute-Common Terminology Criteria for Adverse Events (NCI-CTCAE) Version 4.03. The occurrence of any of the following toxicities during cycle 1 (21 days) was considered a DLT unless it can definitely be attributed to disease progression or other extraneous causes:

Non-hematologic:

1. ≥Grade 4 AEs.
2. Grade 3 immune-related adverse events (AEs).
3. Grade 3 toxicities irrespective of duration, except for the following situations: laboratory abnormalities must not be clinically complicated; diarrhea, nausea, and vomiting that resolved within 3 days.
4. Any grade 3 tumor flare reaction for 7 continuous days or above (local pain, irritation, or rash at known or suspected tumor focus).
5. Abnormalities that satisfy Hy’s Law, i.e., alanine transaminase (ALT) or aspartate transaminase (AST) elevation >3 × upper limit of normal (ULN), total bilirubin elevation >2 × ULN, absence of initial findings of cholestasis (i.e., absence of elevation of alkaline phosphatase to >2 × ULN), and no other reason can be found to explain the combination of increased ALT/AST and total bilirubin.

Hematologic:

1. Grade 4 neutropenia lasting >7 days.
2. Febrile neutropenia (absolute neutrophil count <1000/mm^3^, with a single temperature of 38.3˚C or a sustained temperature of ≥38˚C for more than 1 h).
3. Grade 3 neutropenia with infection.
4. Grade 3 thrombocytopenia with bleeding.
5. Grade 4 thrombocytopenia.
6. Grade 4 anemia (life-threatening).

Any AE of the specified grade and toxicity was a DLT if it could not definitively be attributed to disease progression or other extraneous causes.

Any death not definitively attributed to disease progression or other extraneous causes was a DLT.

Any toxicity that required discontinuation was a DLT.

Patients who received <80% of the nofazinlimab infusion in cycle 1 (e.g., dosing discontinued due to infusion reaction) and did not experience a DLT were not taken into account in the assessment of the overall DLT rate for the particular dose level cohort and were replaced.

DLT for part 2a

All toxicities will be graded according to the NCI-CTCAE Version 4.03. The occurrence of any of the following toxicities during cycle 1 (28 days) will be considered a DLT unless it can definitely be attributed to disease progression or other extraneous cause:

Non-hematologic:

1. ≥Grade 4 AEs.
2. Grade 3 immune-related AEs.
3. Grade 3 toxicities irrespective of duration, except for the following situations: laboratory abnormalities must not be clinically complicated; diarrhea, nausea, and vomiting that resolved within 3 days; grade 3 hand-foot skin reaction, rash (including immune-related rash) and hypertension improve to grade 1 with appropriate medical management within 7 days.
4. Any grade 3 tumor flare reaction for continuous 7 days or above (local pain, irritation or rash at known or suspected tumor focus).
5. Abnormalities that satisfy Hy’s Law, i.e., ALT or AST elevation >3 × ULN, total bilirubin elevation >2 × ULN, absence of initial findings of cholestasis (i.e., absence of elevation of alkaline phosphatase to >2 × ULN), and no other reason can be found to explain the combination of increased ALT/AST and total bilirubin.

Hematologic:

1. Grade 4 neutropenia lasting >7 days.
2. Febrile neutropenia (absolute neutrophil count <1000/mm^3^, with a single temperature of ≥38.3˚C or a sustained temperature of ≥38˚C for more than 1 h).
3. Grade 3 neutropenia with infection.
4. Grade 3 thrombocytopenia with bleeding.
5. Grade 4 thrombocytopenia.
6. Grade 4 anemia (life-threatening).

Any AE of the specified grade and toxicity was a DLT if it cannot definitively be attributed to disease progression or other extraneous causes.

Any death not definitively attributed to disease progression or other extraneous causes was a DLT.

In addition to the criteria listed above, the following toxicities may be declared a DLT after thorough consultation between the investigator and the sponsor:

1. Inability to complete 75% of the prescribed dose of regorafenib due to drug-related AEs.

2. Other drug-related toxicities of any grade that, in the judgment of the investigators and sponsor, are deemed a DLT from a benefit/risk perspective.

3. Inability to start cycle 2 due to a treatment-related AE within 7 days.

Any toxicity that requires discontinuation was considered a DLT.

**Inclusion criteria:**

Patients who met the following criteria as stated in the protocol were included in the study:

1. Willing and able to provide written informed consent for the trial.
2. Male or female and ≥18 years of age on the day of signing informed consent.
3. **Part 1**: Patients with histologically or cytologically confirmed advanced or metastatic tumor (unresectable) must have received, refused, or be intolerant to all available approved or standard therapies known to confirm clinical benefit:
   - Phase 1a: Patients with advanced, relapsed, or refractory solid tumors, which should have referred to but not been limited to the following description for phase 1b.
   - Phase 1b: Patients with tumor of specific types:
     1. For arm 1: Patients with soft tissue sarcoma, including, but not limited to, undifferentiated pleomorphic sarcoma and dedifferentiated or other high-grade liposarcoma.
     2. For arm 2: Patients with malignant pleural mesothelial.
     3. For arms 3 and 4: Patients with bladder cancer, Merkel-cell carcinoma, gastric cancer, esophageal carcinoma, small-cell lung cancer, large-cell lung cancer, head, and neck squamous cell carcinoma, or cutaneous squamous cell carcinoma. Any solid tumors with microsatellite instability-high (MSI-H) or deficient mismatch repair (dMMR). Other tumor types after discussion with the medical monitor and in consultation with the sponsor.
4. **Part 2**: Patients with mCRC who failed at least two lines of standard therapies, including chemotherapies of fluorouracil, oxaliplatin, and irinotecan. Failed therapies were defined as the occurrence of progressive disease or intolerable toxicities during the treatment or within 3 months thereafter the last dose of standard therapy.

Note: 1) One or more chemotherapy drugs listed above should have been used for ≥1 cycle to be considered 1 line of treatment. 2) All three chemotherapy drugs should have been used. 3) Previous adjuvant/neoadjuvant therapy was allowed. If relapse or metastasis occurred during the adjuvant/neoadjuvant treatment period or within 6 months after the completion of the treatment, that adjuvant/neoadjuvant therapy was considered as the failure of first-line systemic therapy. 4) Previous anti-tumor treatment regimens, including chemotherapy combined with epidermal growth factor receptor inhibitors or vascular endothelial growth factor inhibitors, were allowed.

1. Eastern Cooperative Oncology Group Performance Status of 0 or 1.
2. For part 1 phase 1a and part 2a, patients with evaluable but non-measurable lesions were allowed. For part 1 phase 1b and part 2b, patients must have had at least one measurable lesion per RECIST Version 1.1.
3. Tumor tissue samples needed to be collected from the patients for biomarker analysis (optional for part 2a). The samples could have been formalin-fixed, paraffin‑embedded tumor tissue blocks or at least 20 unstained slides. Patients who were not able to provide archival tumor tissues had to be willing to undergo baseline biopsy of the tumor (in quantities determined in accordance with the biopsy results).

Note: Patients were permitted to enroll on a case-by-case basis after discussion with the medical monitor and in consultation with the sponsor if tissue or biopsy was not available.

1. Patients with life expectancy ≥3 months.
2. Patient must have had adequate organ function as indicated by the following laboratory values (had not received blood transfusion, erythropoietin, granulocyte‑colony stimulating factor, or other medical support within 14 days before the administration of the study drug):

| **System** | **Laboratory Value** |
| --- | --- |
| **Hematological** | |
| Absolute neutrophil count | ≥1,500/μL |
| Platelets | ≥100,000/μL |
| Hemoglobin | ≥9 g/dL or ≥5.6 mmol/L |
| **Renal** | |
| Serum creatinine | ≤1.5 × ULN |
| Creatinine clearance | ≥30 mL/min (calculated using Cockcroft-Gault formula) |
| **Hepatic** | |
| Total bilirubin | ≤1.5 × ULN |
| AST (SGOT) and ALT (SGPT) | part 1 phase 1a and part 2a: ≤2.5 × ULN  part 1 phase 1b and part 2b: ≤2.5 × ULN OR  ≤5 × ULN for patients with liver metastases |
| **Coagulation** | |
| INR or PT | ≤1.5 × ULN |
| aPTT | ≤1.5 × ULN |

Abbreviations: aPTT, activated partial thromboplastin time; AST/SGPT, alanine aminotransferase; AST/SGOT, aspartate aminotransferase; INR, international normalized ratio; PT, prothrombin time; ULN, upper limit of normal

1. Fertile men and women of childbearing potential must have agreed to use an effective method of birth control from providing signed consent and for 180 days after last study drug administration. Women of childbearing potential included premenopausal women and women within the first 2 years of the onset of menopause. Women of childbearing potential must have had a negative pregnancy test ≤7 days prior to the first dose of study drug.

**Exclusion criteria:**

Patients who met any of the following criteria as stated in the protocol were excluded from the study:

1. Patients with known brain or central nervous system metastasis that was either symptomatic or untreated. Central nervous system metastases that had been treated by complete resection and/or radiotherapy, demonstrating stability or improvement were not an exclusion criterion provided they were stable as shown by imaging for at least 4 weeks before screening without evidence of cerebral edema and no requirements for corticosteroids or anticonvulsants.
2. Patients with active autoimmune diseases or a history of autoimmune diseases were excluded; these included but were not limited to, patients with a history of immune-related neurologic disease, multiple sclerosis, autoimmune (demyelinating) neuropathy, Guillain-Barre syndrome, myasthenia gravis, systemic lupus erythematosus, connective tissue diseases, scleroderma, inflammatory bowel disease including Crohn’s disease and ulcerative colitis, hepatitis, toxic epidermal necrolysis, Stevens-Johnson syndrome, or antiphospholipid syndrome.

Note: Patients were permitted to enroll if they had vitiligo, eczema, type I diabetes mellitus, or endocrine deficiencies, such as thyroiditis managed with replacement hormones, including physiologic corticosteroids. Patients with rheumatoid arthritis and other arthropathies, Sjogren’s syndrome, controlled celiac disease, and psoriasis controlled with topical medication, and patients with positive serology, such as antinuclear antibodies or antithyroid antibodies should have been evaluated for the presence of target organ involvement and the potential need for systemic treatment but were otherwise eligible.

1. Patients who received glucocorticoids (prednisone at >10 mg/day or other similar drugs at equivalent dose) or other immunosuppressive medication within 14 days prior to the first dose of the study drug.

Note: Adrenal replacement doses ≤10 mg daily prednisone equivalents were permitted in the absence of active autoimmune disease; patients were permitted to use topical, ocular, intra-articular, intranasal, and inhalational corticosteroids (with minimal systemic absorption); a brief course of corticosteroids for prophylaxis (e.g., contrast dye allergy) or for treatment of nonautoimmune conditions (e.g., delayed-type hypersensitivity reaction caused by contact allergen) was permitted.

1. Part 1 phase 1b and part 2b patients who had other malignant tumor (s) in the past 2 years, except for patients with basal cell carcinoma, in situ breast cancer, and cervical carcinoma in situ who had undergone radical treatment.
2. Patients who received any targeted T cell coregulated proteins (immune checkpoint proteins) antibody/medicine (including PD‑1, PD‑L1) for treatment.
3. Part 2:

- Patients with known MSI-H/dMMR.
- Patients who previously received regorafenib, fruquintinib, and other vascular endothelial growth factor receptor tyrosine kinase inhibitors.
- Patients who received potent CYP3A4 inhibitors or inducers prior to the first dose of the study drug (the patient could be enrolled if the elution period prior to the first dose of the study drug was ≥5 half-lives) or patients who needed to continue receiving these medications during the study period.
- Patients with uncontrolled hypertension: systolic blood pressure >140 mmHg or diastolic pressure >90 mmHg despite optimal medical management.
- Patients with any hemorrhage or bleeding event ≥ grade 3 (CTCAE) within 28 days prior to the start of study treatment.
- Patients unable to swallow oral medications or with any malabsorption condition.

1. Patients who had prior chemotherapy, targeted therapy, or any other agents used as systemic treatment for cancer within 2 weeks prior to the first dose of the study drug.
2. Patients who had undergone a major surgical procedure (as defined by the investigator) or wide field of radiation within 28 days prior to the first dose of the study drug or received local radiotherapy within 14 days prior to the first dose of the study drug, or taken radioactive agents (e.g., strontium, samarium) within 56 days before the first dose of study drug.
3. Patients who had received treatment with any herbal or alternative therapies or Chinese prepared medicine within 7 days prior to the first dose of the study drug.
4. Patients who had received a live and attenuated vaccine within 28 days prior to the first dose of the study drug.
5. Patients who had a history of interstitial lung disease or noninfectious pneumonitis except for those induced by radiation therapies.
6. Patients who had a known history of HIV infection.
7. Patients who were hepatitis B surface antigen and hepatitis B core antibody positive or hepatitis C virus (HCV) antibody positive at screening must not have been enrolled until further definitive testing with hepatitis B virus (HBV) DNA titers, and HCV RNA tests conclusively ruled out the presence of active infection (HBV DNA ≥1000 cps/mL or 200 IU/mL) that required therapy with hepatitis B and C, respectively.
8. Patients who had an active infection of tuberculosis.
9. Patients who had signs or symptoms of any active infection requiring systemic therapy.
10. Patients who had received organ transplantation.
11. Patients who had any unresolved CTCAE grade ≥2 toxicities from prior anti-cancer therapy with the exception of vitiligo, alopecia, and the laboratory values defined in the inclusion criteria.
12. Patients who had a history of any immune-related AE of grade ≥3.
13. Patients who had a serious hypersensitive reaction to monoclonal antibodies and had a history of uncontrolled allergic asthma.
14. Patients with a known history of alcoholism or drugs abuse.
15. Patients with major cardiovascular diseases (e.g., congestive heart failure, unstable angina pectoris, atrial fibrillation, arrhythmia): patients who had experienced such diseases as acute myocardial infarction, unstable angina pectoris, apoplexia, or transient ischemic attack within 6 months prior to the first dose of study drug; patients with congestive heart failure of New York Heart Association grade ≥2.
16. Patients who had known psychiatric disorders that would interfere with cooperation with the requirements of the trial.
17. Patients who had a concurrent condition that, in the investigator’s opinion, would have jeopardized compliance with the protocol.
